# PhytoCRISP-Ex: a web-based and stand-alone application to find specific target sequences for CRISPR/CAS editing

**DOI:** 10.1186/s12859-016-1143-1

**Published:** 2016-07-01

**Authors:** Achal Rastogi, Omer Murik, Chris Bowler, Leila Tirichine

**Affiliations:** Institut de Biologie de l’Ecole Normale Supérieure (IBENS), Ecole Normale Supérieure, PSL Research University, CNRS UMR 8197, INSERM U1024, 46 rue d’Ulm, F-75005 Paris, France

**Keywords:** CRISPR, Cas9, Protists, Genome editing, Eukaryotes

## Abstract

**Background:**

With the emerging interest in phytoplankton research, the need to establish genetic tools for the functional characterization of genes is indispensable. The CRISPR/Cas9 system is now well recognized as an efficient and accurate reverse genetic tool for genome editing. Several computational tools have been published allowing researchers to find candidate target sequences for the engineering of the CRISPR vectors, while searching possible off-targets for the predicted candidates. These tools provide built-in genome databases of common model organisms that are used for CRISPR target prediction. Although their predictions are highly sensitive, the applicability to non-model genomes, most notably protists, makes their design inadequate. This motivated us to design a new CRISPR target finding tool, PhytoCRISP-Ex. Our software offers CRIPSR target predictions using an extended list of phytoplankton genomes and also delivers a user-friendly standalone application that can be used for any genome.

**Results:**

The software attempts to integrate, for the first time, most available phytoplankton genomes information and provide a web-based platform for Cas9 target prediction within them with high sensitivity. By offering a standalone version, PhytoCRISP-Ex maintains an independence to be used with any organism and widens its applicability in high throughput pipelines. PhytoCRISP-Ex out pars all the existing tools by computing the availability of restriction sites over the most probable Cas9 cleavage sites, which can be ideal for mutant screens.

**Conclusions:**

PhytoCRISP-Ex is a simple, fast and accurate web interface with 13 pre-indexed and presently updating phytoplankton genomes. The software was also designed as a UNIX-based standalone application that allows the user to search for target sequences in the genomes of a variety of other species.

**Electronic supplementary material:**

The online version of this article (doi:10.1186/s12859-016-1143-1) contains supplementary material, which is available to authorized users.

## Background

Phytoplankton are microalgae that form an essential constituent of the marine food chain. Though microscopic and mostly uncharacterized, these minute organisms have tremendously showcased themselves as potential research models [22, 20]. Recent large-scale sampling to understand the morphological and genetic diversity of this hidden community [3, 12, 21] has already established the foundation for further molecular studies. The successful achievement of this exploration also reflects the interest of research communities towards the functional characterization of phytoplankton in the near future.

Clustered regularly interspaced short palindromic repeats CRISPR/CAS systems have recently emerged as a simple and accurate tool for genome editing [13], and show facile editing in numerous organisms including bacteria [10], yeast [4], plants [6], human [14] and other animals [2, 5, 7, 9, 17, 23, 24]. Common designed CRISPR systems consist of expression of a Cas9 nuclease or nickase and a single guide RNA (sgRNA). The latter includes a 20-bp target sequence used to target the Cas9/RNA complex to the desired chromosomal location. By modifying only these 20-bp, the targeting of the whole CRISPR/Cas9 complex will change and thus the DNA cleavage site. A well designed target sequence must not only optimally bind to its desired target, but must also not target any other site in the genome being edited, to avoid undesired off-target mutations [11]. Several parameters were shown to contribute to a better targeting of the target sequence, and the general form of G-N19-NGG is widely accepted, although others were also suggested [1].

Because target site choice is a key point for promoting successful editing, several computational tools have been designed aiming to automate this procedure, all offering candidate target sites for a given input sequence/gene and potential off-target sites for a given background genome. This search is usually restricted for genomes of selected model organisms such as human, mice, fruit-fly, *C. elegans*, and yeast, making them irrelevant for researchers aiming to use CRISPR on other, less common, organisms. We designed PhytoCRISP-Ex, a user friendly web interface, as well as a stand-alone software, which predicts potential target sites for CRSIPR/CAS projects. The off-target analysis can be performed against indexed genomes from 13 algae (diatoms, green algae, haptophytes, etc.), or any user defined genome or transcriptome, assembled or not, making it useful for designing CRISPR projects for many communities.

### Algorithm

**Fig. 1:**
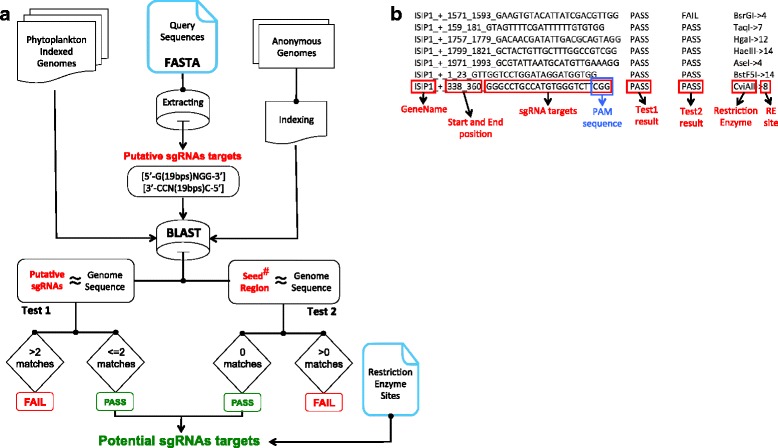
PhytoCRISPex workflow. The figure represents (**a**) the working design of PhytoCRISPex, taking NGG as a PAM sequence and (**b**) the description of sample PhytoCRISPex output file. **a** The flowchart here demonstrates the working of the web server and the standalone version of the software. The genome is indexed and putative sgRNA targets (structured as [5′-G/N(19 bps)NGG/NAG-3′]/[3′-CCN/CTN(19 bps)C/N-5′]) from user given query sequences are aligned locally against it. The aligned output is then directed through two check levels, passing both assigns a putative sgRNA target as potential target for cas9 activity. **b** The output file of the software is a comma separated file which can be viewed easily using Excel. The file includes three basic columns. The first column represents gene name with start, stop and sequence of the sgRNA targets. The next two columns are the results of check level one and two, respectively

PhytoCRISP-Ex is a rational and flexible tool for finding Cas9 target sites with low/no off-target potential. Given a DNA query (e.g., gene) sequence, the pipeline first fetches all the possible regions of length 23, structured as [5′-G/N(19 bps)PAM-3′]**/**[3′-PAM(19 bps)C/N-5′]. These regions are then evaluated and filtered to show low/no off-target activity across the whole genome (see Fig. 1a for general workflow). The choice of PAM sequence and CRISPR target start base is kept flexible. PAM sequence can be selected from NGG or NAG and CRISPR start base can be chosen as G or any (N) base. These options are also implemented in the standalone version of the software and can be used as arguments.

There are two levels of filters and passing both designates the region as a potential sgRNA for Cas9 activity. The first level accepts the target sequence and tags it “PASS” if it has less than or equal to 2 base-pair mismatch with the closest off-target in the genome. The second filter, accepts the target if the seed region (last 15 bases including the PAM sequence) has 0 mismatch with off-targets anywhere in the genome. The pipeline reports the instances which are accepted by both or either one of the filters, but the ones which passes both the filters are designated as potential Cas9 targets.

Once the potential sgRNAs are filtered, they are then checked for the presence of none, one or more restriction sites corresponding to pre-selected and most common restriction enzymes (Additional file 1: Figure S1). Cas9 enzyme cleaves both DNA strands ~3–4 bases upstream of the PAM [19, 25]. Therefore along with the presence of restriction sites on the entire target sequence, PhytoCRISP-Ex reports specifically, if any, the restriction sites overlapping three and four bases upstream to the PAM sequence. Embedding a restriction site in the target sequence will help screening for mutants using PCR after a digest with the appropriate restriction enzyme. Presence and absence of the restriction sites along with its position on the sgRNA are reported in the output file (Fig. 1b). The list of restriction enzymes (Additional file 2: Table S1) can be updated and used when using the standalone version of the software. The PhytoCRISP-Ex pipeline is mounted with the index of 13 genomes and is accessible via web-server. An off-line standalone software is also developed which gives liberty to its users to use it for any genome, assembled or un-assembled. The standalone package also includes a few example files and a README file to help users install and execute PhytoCRISP-Ex. The standalone version can be found under “Download” tab at http://www.phytocrispex.biologie.ens.fr/CRISP-Ex/.

## Results

Several computational tools have been published aiming to identify candidates for CRISPR/CAS targeting. In order to validate the novelty and sustainability of PhytoCRISP-Ex, we compared it with 8 other previously published tools. Our criteria for comparison included first, the possibility to use the tool as a browser-based or stand-alone application, so to meet the need of a wide range of users. Second, whether it provides the flexibility to perform off-target analysis on any or a restricted list of genomes. The third criteria compares the possibility to perform restriction site mutant screening analysis. The summary of this comparison is presented in Table 1 and shows PhytoCRISP-Ex as a robust and adequately designed application. Further, we evaluated our tool against the whole exome of the model diatom *Phaeodactylum tricornutum* (http://protists.ensembl.org/Phaeodactylum_tricornutum/Info/Index/) and *Thalassiosira pseudonana* (http://genome.jgi.doe.gov/Thaps3/Thaps3.home.html)*.* The statistics revealed that ~94 % and ~95 % of the genes in *P. tricornutum* and *T. pseudonana*, respectively, have atleast 1 potential guide RNA to be used as a Cas9 target against these genes. Among the latter, most of the genes have high percent efficiency in terms of constituting mostly potential targets among total predicted Cas9 targets (Additional file 1: Figure S1). These findings suggest that potentially almost any gene in these two species can be targeted with high probability for generating a single specific mutation. Out of the total, ~89 % genes in both the species (*P. tricornutum* and *T. pseudonana*) possess potential targets with restriction sites over the probable Cas9 cleavage site. Therefore, choosing such targets might help in fast screening of the mutants. PhytoCRISP-Ex is a CRISPR/Cas9 target extraction web-package, built to extract targets using 13 model phytoplankton genomes. The algorithm has also been designed as a UNIX based standalone package which provides flexibility to its users to use it on other non-model genomes. The simple design of PhytoCRISP-Ex allows its use by end-users with moderate or no software programming background.

**Table 1:** PhytoCRISP-Ex vs others

Softwares	Browser-based application	Stand-alone application	Restriction screening	Background Genome flexibility	Reference
PhytoCRISP-Ex	✓	✓	✓	✓	Current study
CRISPR MultiTargeter	✓	X	X	X	[18]
CasFinder	X	✓	X	X	[1]
CHOPCHOP	✓	X	X	X	[15]
CRISPRdirect	✓	X	X	X	[16]
E-CRISP	✓	X	X	X	[8]
sgRNAcas9	X	✓	X	✓	[27]
CasOT	X	✓	X	✓	[26]
CRISPRseek	X	✓	✓	✓	[28]

Comparison between PhytoCRISP-Ex and several previously published CRISPR target analysis tools

## Conclusions

With the persuasive interest of scientific community towards phytoplankton research, the need of establishing genetic transformation tools for plankton species is thriving. PhytoCRISP-Ex provides a reliable and first ever application for predicting CRISPR/Cas9 targets within various plankton genomes. PhytoCRISP-Ex is also equipped with an easy to use, yet powerful, standalone version which gives its user the flexibility to use it on any genome. Many such and other unique features make the software more advance and appropriate for the use by a broad research community.

## Abbreviations

CAS, CRISPR associated protein; CRISPR, Clustered regularly-interspaced short palindromic repeats; DNA, Deoxyribo nucleic acid; PAM, Protospacer adjacent motif; PCR, Polymerase chain reaction; RNA, Ribo nucleic acid.

## Additional files

Additional file 1: Figure S1. PhytoCRISP-Ex efficacy. The scatter plot depicts that most of the predicted Cas9 targets per gene in *Thalassiosira pseudonana* and *Phaeodactylum tricornutum*, respectively, are potential candidates (passing both PhytoCRISP-Ex filter). X-axis represents the percent efficiency of each gene in terms of having high number of potential Cas9 targets compared to the total number of targets. Y-axis represents the number of all potential targets per gene. (PDF 182 kb) Additional file 2: Table S1. The list of restriction enzymes being used by PhytoCRISP-Ex. (XLSX 13 kb)
