# Supplementary figures and images for: PhytoCRISP-Ex: a web-based and stand-alone application to find specific target sequences for CRISPR/CAS editing
Source: BMC Bioinformatics. 2016 Jul 1;17:261. doi: 10.1186/s12859-016-1143-1 (PMC4929763; doi:10.1186/s12859-016-1143-1)

### *Thalassiosira pseudonana*

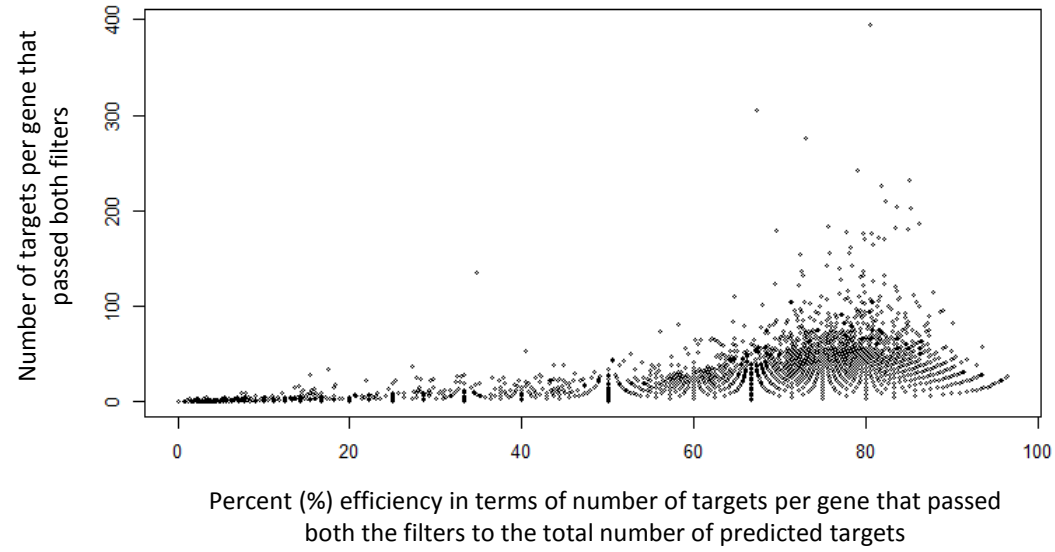

### *Phaeodactylum tricornutum*

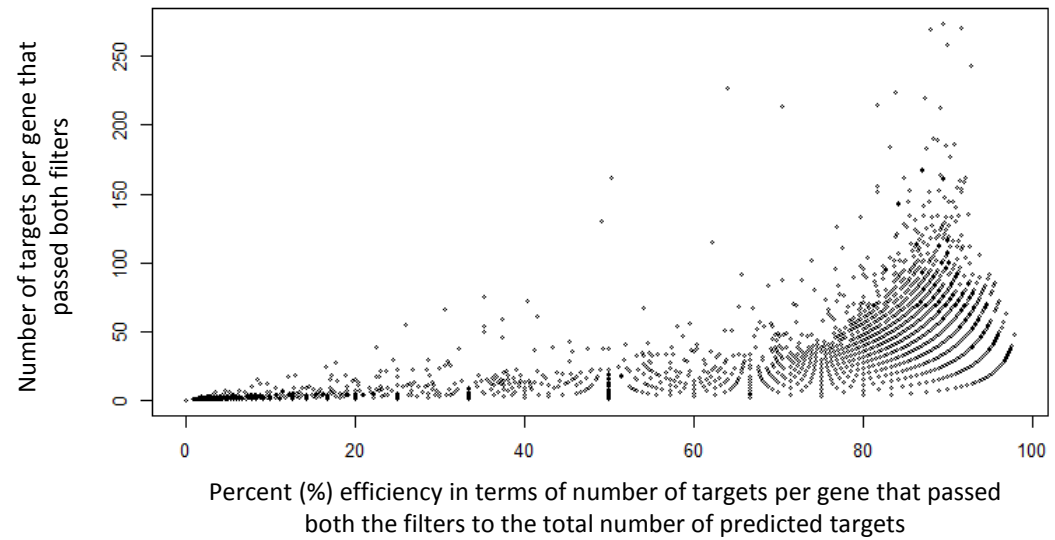

Supplement: Additional file 1: Figure S1. — PhytoCRISP-Ex efficacy. The scatter plot depicts that most of the predicted Cas9 targets per gene in Thalassiosira pseudonana and Phaeodactylum tricornutum, respectively, are potential candidates (passing both PhytoCRISP-Ex filter). X-axis represents the percent efficiency of each gene in terms of having high number of potential Cas9 targets compared to the total number of targets. Y-axis represents the number of all potential targets per gene. (PDF 182 kb) [file 12859_2016_1143_MOESM1_ESM.pdf]
